# Prospective longitudinal study on quality of life in relapsed/refractory multiple myeloma patients receiving second- or third-line lenalidomide or bortezomib treatment

**DOI:** 10.1038/bcj.2017.20

**Published:** 2017-03-17

**Authors:** X Leleu, C Kyriakou, I Vande Broek, P Murphy, P Bacon, P Lewis, H Gilet, B Arnould, M T Petrucci

**Affiliations:** 1 Hopital de La Milétrie - CHU and CIC Inserm 1402, Poitiers, France; 2 Royal Free and Northwick Park Hospitals, London, UK; 3 Iridium Kankernetwerk, Sint-Niklaas, Belgium; 4 Beaumont Hospital, Dublin, Ireland; 5 Celgene International Sarl, Boudry, Switzerland; 6 Celgene GmbH, Munich, Germany; 7 Patient-Centered Outcomes, Mapi, Lyon, France; 8 Division of Hematology, Department of Cellular Biotechnology and Hematology, Sapienza University of Rome, Rome, Italy

## Abstract

Treatment advances for multiple myeloma (MM) that have prolonged survival emphasise the importance of measuring patients' health-related quality of life (HRQoL) in clinical studies. HRQoL/functioning and symptoms of patients with relapsed/refractory MM (RRMM) receiving second- or third-line lenalidomide or bortezomib treatment were measured in a prospective European multicentre, observational study at different time points. At baseline, patients in the lenalidomide cohort were frailer than in the bortezomib cohort with more rapid disease progression at study entry (more patients with Eastern Cooperative Oncology Group performance status >2, shorter time from diagnosis, more chronic heart failure, higher serum creatinine levels, more patients with dialysis required). About 40% of the patients receiving lenalidomide discontinued the study in <6 months while 55% in the bortezomib cohort discontinued. No substantial HRQoL deterioration was observed for the first 6 months in patients with RRMM receiving one or the other treatment. For patients still on treatment at study completion (month 6), only the European Organization for Research and Treatment of Cancer Quality-of-Life Core domains of Diarrhoea and Global Health Status/QoL had worsened in the lenalidomide and bortezomib cohorts, respectively. A clinically meaningful deterioration in HRQoL was more often observed for patients who discontinued the study prior to 6 months in the bortezomib cohort than in the lenalidomide cohort.

## Introduction

Multiple myeloma (MM) is an incurable haematological malignancy characterised by bone marrow infiltration of malignant plasma cells leading to impaired haematopoiesis, immunosuppression and a high incidence of bone lesions that can cause pathologic fractures and severe bone pain.^1, 2^ MM accounts for 10% of the malignant haematological diseases and approximately 1% of all cancer-related deaths in Western countries.^3^ It was estimated that, in 2015, 26 850 new cases of MM would be diagnosed and 11 240 patients would die from the disease in the United States of America.^4^ In Europe, MM is diagnosed in approximately 38 956 patients and claims about 24 296 lives each year.^3^

Despite the considerable improvements in the treatment, the majority of the MM patients will experience multiple subsequent relapses of their disease requiring subsequent treatment.^5^ There are increasingly more novel agent options in managing MM at diagnosis and relapse,^6^ including the proteasome inhibitors bortezomib, carfilzomib and recently US Food and Drug Administration-approved ixazomib,^7, 8^ the immunomodulatory drugs pomalidomide, lenalidomide and thalidomide and recently Food and Drug Administration-approved daratumumab and elotuzumab.^8, 9^ However, at some point MM can become refractory following multiple lines of treatment and resistant to the currently available therapies.^10^ While MM has no cure, successive lines of treatment can lead to greater risk of developing adverse reactions that could be in turn responsible for sequelae and create handicap impacting patients' quality of life.

Health-related quality of life (HRQoL) is a multidomain concept that represents the patient's perception of the effect of illness and treatment on physical, psychological and social aspects of life.^11^ Assessing HRQoL is critical to better capture health aspects that matter to the patients themselves and that go beyond the prolongation of life.^12^

Among current treatments used, intravenously or subcutaneously administered bortezomib and orally administered lenalidomide showed statistically significant improvements in phase III trials for the treatment of relapsed/refractory MM (RRMM) in terms of overall response rate, complete response rate, time to progression and overall survival.^13, 14^ However, even though a large body of evidence supports the clinical benefits of lenalidomide^13, 15, 16^ and bortezomib^14, 17^ for RRMM patients, the literature is relatively scarce regarding the burden that treatment poses on patients' HRQoL as reported directly by patients.^18, 19^

In order to better understand how treatments for RRMM impact patients in terms of HRQoL in the real-life context, a European, prospective, multicentre, non-interventional, longitudinal study was conducted in RRMM patients beginning second- or third-line treatment with either lenalidomide or bortezomib, the two RRMM treatment options available at the time of study implementation. The objective of this study is to describe and better understand patients' HRQoL when treated with bortezomib or lenalidomide for RRMM.

## Materials and methods

### Patients

Patients were eligible for study enrolment if they had RRMM requiring second- or third-line treatment with either lenalidomide- or bortezomib-based regimens and with at least one measurable disease manifestation: any quantifiable serum monoclonal protein value (generally, but not exclusively, >1 g/dl immunoglobulin G M-protein or >0.5 g/dl immunoglobulin A) and, where applicable, urine light-chain excretion of ⩾200 mg/24 h + presence of soft tissue (not bone) plasmacytomas as determined by clinical examination or applicable radiographs (that is, magnetic resonance imaging, computed tomographic scan) or a quantifiable plasma cell infiltration of the bone marrow as determined by bone marrow biopsy. Patients who were planned to receive a stem-cell transplant as part of the second-line treatment for MM and patients who were treated with a cytotoxic drug in combination with lenalidomide or bortezomib were excluded from the study.

This study was conducted in compliance with the Declaration of Helsinki and all current national regulations. In accordance to local requirements, the study protocol was reviewed and approved by the Independent Ethics Committee prior to the inclusion of patients into the study. All patients gave written informed consent prior to their inclusion.

### Study design

This was a European, prospective, multicentre, observational, longitudinal study conducted in six countries (Belgium, France, Germany, Ireland, Italy and United Kingdom). Patients were identified by their physician either through a prescreening and/or during the course of routine patient visits. Recruited patients were followed up for a maximum of 6 months. Physicians were asked to specify the reason for any patients not completing the study: disease progression, discontinuation of treatment, or any other reasons, including death, withdrawn consent or lost to follow-up.

Socio-demographic and clinical data were collected at baseline by the recruiting physicians. HRQoL was assessed using patient-completed questionnaires at baseline, 3 months and 6 months following treatment initiation and/or at study discontinuation.

### Assessments

HRQoL was assessed using three questionnaires, the European Organization for Research and Treatment of Cancer (EORTC) Quality-of-Life Core (QLQ-C30),^20, 21^ QLQ-Multiple Myeloma (QLQ-MY20)^22, 23^ and QLQ-Chemotherapy-Induced Peripheral Neuropathy (QLQ-CIPN20)^24^ instruments. The QLQ-C30 includes 30 items distributed across six Functional domains (Cognitive, Emotional, Physical, Role and social functioning, Global health status/QoL) and nine Symptom domains (Appetite loss, Constipation, Diarrhoea, Dyspnoea, Fatigue, Financial difficulties, Nausea and vomiting, Pain, Insomnia). The QLQ-MY20 includes 20 items distributed across two Functional domains (Body image, Future perspective) and two Symptom domains (Disease symptoms, Side effects of treatment). The QLQ-CIPN20 includes 20 items distributed across three symptom domains (Autonomic scale, Motor scale, Sensory scale). For all questionnaires, all items were answered on a four-point Likert scale ranging from ‘not at all' to ‘very much', except items 29 and 30 of QLQ-C30 that are answered on a seven-point Likert scale ranging from ‘very poor' to ‘excellent'. Scores were converted to a range from 0 to 100; for Functional domains, higher scores indicate better functioning; for Symptom domains, higher scores indicate greater symptom.

### Statistical analysis

Descriptive statistics were applied to describe the population, the individual components of the EORTC questionnaire and the change in EORTC questionnaire scores from baseline to month 3, month 6 or study discontinuation. Changes in EORTC scores were calculated only for patients who were able to complete the questionnaires at both baseline and the time point of interest.

Minimal important difference (MID), defined as the smallest change in a quality of life score considered important to patients, was estimated within the study to provide support for the interpretation of changes in scores.^25^ MID was calculated as 0.5 × SD_bas_, with SD_bas_ the s.d. of the score at baseline, for single-item domains, and as the s.e.m., defined as SD_bas_ × (1−*r*)^1/2^ with *r* the Cronbach's alpha reliability coefficient, for multi-item domains.^26^

Patients in the bortezomib cohort were described by standard- or low-dose treatment: bortezomib treatment was considered as standard dose if ⩾6 vials and as low dose if <6 vials within a 6-week period, a vial corresponding to an injection of dose of 1.3 mg/m^2^ body surface area as per the summary of product characteristics.

The statistical analysis was performed using the SAS software for Windows (Version 9.2, SAS Institute Inc., Cary, NC, USA).

## Results

### Study population

#### Patients' disposition and characteristics

**Figure 1:**
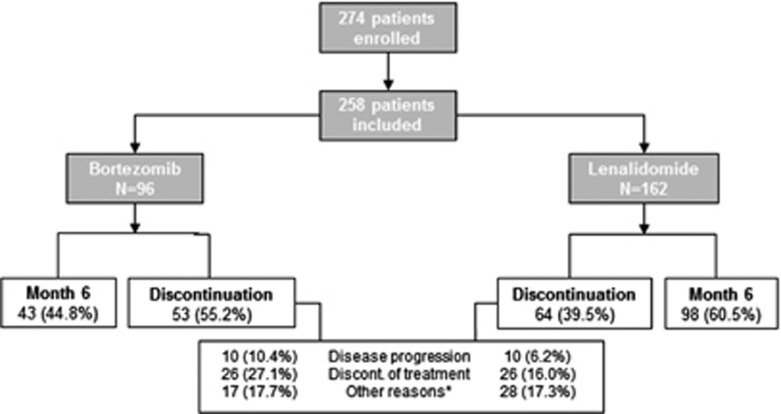
Patients' disposition and study completion by treatment group. Reasons for discontinuation are as reported by the physician. *Other reasons include death, loss to follow-up, withdrawn consent, other and missing (include two patients in the bortezomib cohort who never received treatment: one discontinued for consent withdrawal and the other for other reasons).

Out of the 274 patients enrolled from December 2010 to February 2014 by 33 sites, 258 (94.2%) patients met the selection criteria and were included in the study. More patients initiated a treatment with oral lenalidomide (*n*=162) than with injectable bortezomib (*n*=96) (Figure 1). Among the 96 patients receiving bortezomib, 41 (42.7%) had the drug administered intravenously and 11 (11.5%) subcutaneously; the data were missing for 44 (45.8%) patients. Most patients in both cohorts received concomitant dexamethasone. Some differences in baseline characteristics (Table 1) were observed between the lenalidomide and bortezomib cohorts: the oldest quartile of patients was somewhat older in the lenalidomide cohort (77–93 years) compared with the bortezomib cohort (74–85 years). Also, the proportion of Eastern Cooperative Oncology Group performance status >2 was slightly higher in the lenalidomide cohort (6.2%) vs the bortezomib cohort (3.1%). Time since MM diagnosis was longer by >1 year in the bortezomib cohort (3.9±3.0 years) compared with the lenalidomide cohort (2.8±2.5 years); also the upper quartile of time from diagnosis in the bortezomib cohort was 4.9–20.5 years vs 3.4–12.4 years in the lenalidomide cohort (Table 1). The *ad hoc* statistical analysis showed that age and time since diagnosis were significantly different across the two cohorts (*P*<0.05). This *ad hoc* analysis also showed that requirement for dialysis and treatment line were significantly different across the two cohorts (*P*<0.05).

Although fewer patients in the lenalidomide cohort (13.0%) were diabetic compared with the bortezomib cohort (18.8%), slightly more had chronic heart failure (14.8% vs 8.3%, respectively). Median serum creatinine levels were equal between the lenalidomide and bortezomib cohorts, but the upper quartile in the lenalidomide cohort displayed far higher serum creatinine levels (114–2934 μmol/l) as compared with the bortezomib cohort (106–402 μmol/l). This was also reflected by the fact that 9 patients (5.6%) in the lenalidomide cohort required dialysis vs no patients in the bortezomib cohort. Slightly more patients in the lenalidomide cohort had baseline neuropathic pain (14.8 vs 6.3% in the bortezomib group) (Table 1). Altogether, these characteristics suggest frailer patients in the lenalidomide cohort with a more rapid disease progression at baseline than in the bortezomib cohort.

Out of the 162 patients receiving lenalidomide, 64 (39.5%) discontinued the study before 6 months; out of the 96 patients receiving bortezomib, 53 (55.2%) discontinued the study before 6 months. Reasons for discontinuing the study are shown in Figure 1. Twenty patients (64.5%) in the standard-dose bortezomib cohort discontinued the study and 25 patients (43.9%) in the low-dose bortezomib cohort (data not shown). Eight patients could not be classified as standard- or low-dose bortezomib because of too few bortezomib dosing administrations during the study. Detailed information of the dosage of treatment received during the study is presented in Table 2.

Mean study duration was about 5 months in the lenalidomide cohort and 4 months in the bortezomib standard- and low-dose cohorts.

### Longitudinal HRQoL results for lenalidomide and bortezomib cohorts

EORTC questionnaires were completed at baseline by 93 (96.9%) patients in the bortezomib cohort and 158 (97.5%) patients in the lenalidomide cohort, at month 3 by 65 (100%) and 122 (100%) patients, at month 6 by 43 (100%) and 94 (95.9%) patients and at study discontinuation by 29 (54.7%) and 27 (42.2%) patients in the bortezomib and lenalidomide cohorts, respectively. Baseline HRQoL scores are presented in Table 3.

**Figure 2:**
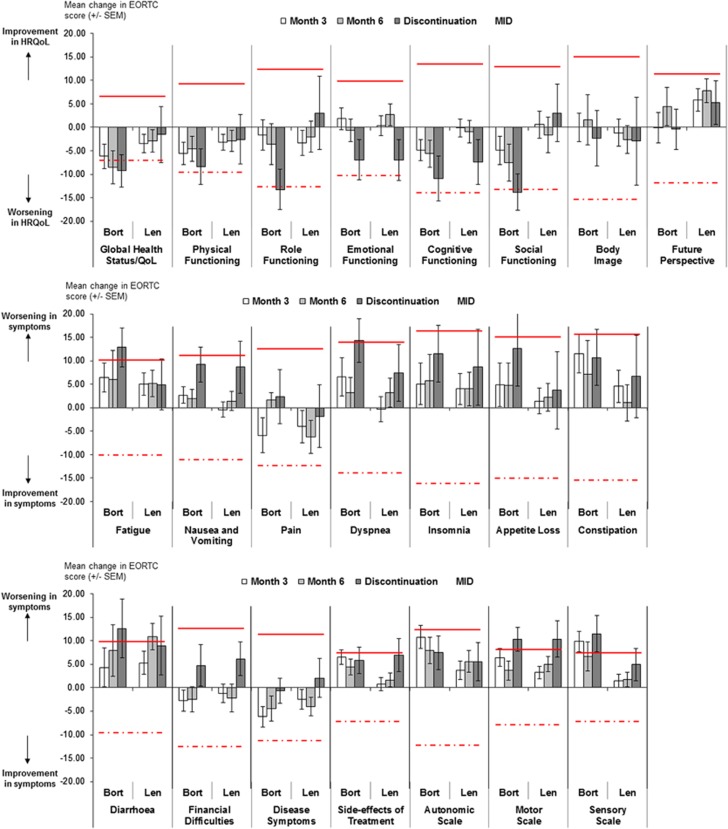
Description of changes in domains scores for the three EORTC questionnaires over time in the lenalidomide and bortezomib cohorts. Bort: bortezomib, with *N*=59–62 at month 3, 40–42 at month 6 and 27–29 at discontinuation; Len: lenalidomide, with *N*=113–120 at month 3, 90–93 at month 6 and 23–27 at discontinuation; MID corresponding to a meaningful worsening (straight line), and a meaningful improvement (dashed line), MID defined as standard error of measurement for multi-item domains and as 0.5 × SD at baseline for single-item domains.

At study completion (month 6), HRQoL reductions from baseline reaching MID were observed for 1 of the 22 domains in each cohort: Diarrhoea domain in the lenalidomide cohort (mean change (s.d.) of 10.9 (27.1), indicating a worsening of the symptom) and Global health status/QoL domain in the bortezomib cohort (mean change (s.d.) of −8.5 (22.7), indicating a worsening of HRQoL). For all other domains, changes over time did not reach the MID. A slight deterioration in HRQoL was consistently observed over time in both lenalidomide and bortezomib cohorts for all other domains, except Financial difficulties, Pain, Disease symptoms and Future perspective domains, where a slight improvement was observed (Figure 2).

For patients who discontinued the study prior to 6 months owing to disease progression or discontinuation of treatment, clinically meaningful declines in HRQoL exceeding the MID were observed in 8 of the 22 domains for the bortezomib cohort (Global health status/QoL, Role functioning, Social functioning, Fatigue, Dyspnoea, Diarrhoea, Motor scale and Sensory scale domains) and in 1 of the 22 domains in the lenalidomide cohort (Motor scale domain) (Figure 2).

## Discussion

The objective of this prospective, observational European 6-month study was to investigate the HRQoL of patients diagnosed with RRMM receiving second- or third-line lenalidomide or bortezomib treatment. In this real-world setting, the results showed that HRQoL was not substantially impaired under continued treatment with either lenalidomide- or bortezomib-based regimens. Only a change indicating a worsening in the Diarrhoea domain from baseline to month 6 was observed in the lenalidomide cohort, and a change indicating a worsening in the Global health status/QoL domain was observed in the bortezomib cohort.

Patients who discontinued therapy early showed worsening of 8 of the 22 EORCT domains in the bortezomib group but only in 1 domain in the lenalidomide group. One can hypothesise that this could be linked to the fact that patients on bortezomib who discontinued early more frequently have symptoms on relapse compared with patients on lenalidomide.

In our study, we did observe some differences in patient populations, but as this is a real-world setting, patient populations should not be directly compared between treatment cohorts. Other considerations are that several factors may impact a physician's decision to administer one treatment, such as: patient's performance status, prior line of therapy, disease characteristics and risk factors, and local national limitations for drug funding approval guidelines.

As an observational study, physician choice could have contributed to the imbalance in the number of patients in each treatment cohort and in dosing schedule for bortezomib with approximately 60% of patients receiving low-dose bortezomib, with a mean (s.d.) average dosage of 3.6 (1.7) mg/m^2^ body surface area per 6-week period.

According to the summary of product characteristics, the recommended treatment duration of bortezomib for RRMM is four cycles followed by four cycles in the case of response or stable disease. In our study, the median duration was approximately 4 months, which is short compared with the label. However, in an Italian retrospective study of 85 patients with second-line therapy, the median treatment duration was 4.1 months;^27^ so it may be the case that the actual duration of treatment is lower than the summary of product characteristics in normal clinical use.

Despite being the second most common haematological cancer,^3^ MM remains rare. In the context of this study conducted with patients with RRMM starting second- or third-line treatment, small sample sizes could be expected in particular when focussing on subgroups of patients at discontinuation. Further research is required to draw more robust conclusions. Differentiation of HRQoL at discontinuation between patients discontinuing the study due to disease progression and due to discontinuation of treatment would also have been interesting, but it could not be made here owing to the small sample size in the subgroups.

In addition to sample size, another limitation of this study is the low number of patients who received subcutaneous bortezomib, now a common form of treatment in RRMM.^28^ This prevented us from drawing meaningful conclusions with regard to QoL between subcutaneously and intravenously administered bortezomib.

Finally, while it is generally recommended using both distribution- and anchor-based approaches and a range of MIDs rather than a unique value, MIDs defined in our study were similar to the ones previously reported by Kvam *et al.*^29^ in 2010 which were defined using values representing minimal changes that patients regard as a definite improvement or deterioration.

This study focusses on RRMM; however, with the approval of lenalidomide in combination with dexamethasone in frontline MM in February 2015 by the European Medicines Agency (EMA),^30^ the question on bortezomib vs lenalidomide has also become relevant for newly diagnosed MM (NDMM) patients. HRQoL at discontinuation due to disease progression was analysed separately from individual HRQoL measurement time points in the FIRST trial comparing lenalidomide in combination with dexamethasone to the combination of melphalan, prednisone and thalidomide in NDMM.^31^ Improvements in HRQoL were generally maintained for patients on continued treatment with lenalidomide–dexamethasone. The VISTA study also investigated HRQoL outcomes in NDMM patients randomised to melphalan–prednisone in combination or not with bortezomib, but there was no distinction made between patients completing the study and patients discontinuing the study early.^32^ Findings on lenalidomide and bortezomib in the context of HRQoL in RRMM, especially at discontinuation, may well be similar in NDMM given the treatments' general mode of action. However, it requires further studies on HRQoL in frontline MM in order to confirm this hypothesis. Also, one cannot exclude that the decreased HRQoL observed upon treatment discontinuation, may be partly a result of disease progression and not only a result of a direct drug effect.

Ultimately, there remains no cure for MM, and while we have made significant advances in the length of time that patients diagnosed with MM are living with the disease, it is important to ensure that with this longevity there is an acceptable quality of life, as reflected in their HRQoL functions. This study has enabled us to observe, in a real-world setting, the impact that continuous treatment over a 6-month period had on the patients' HRQoL scores and showed that they did not substantially deteriorate, despite receiving treatment with associated dverse events that could have potentially impacted patient's well-being. Importantly, some differences in HRQoL deterioration were observed between bortezomib and lenalidomide at the time of discontinuation of treatment.

## Figures and Tables

**Table 1:** Baseline demographic and clinical characteristics

*Variable*	*Bortezomib (*N=*96)*	*Lenalidomide (*N=*162)*	P*-value*^a^
*Age, years*			
Mean (s.d.)	68.0 (9.1)	70.9 (9.8)	**0.022**
Median (Q1–Q3)	69.0 (62.0–74.0)	72.0 (66.0–77.0)	
Min–max	38.0–85.0	29.0–93.0	
			
*Gender, %*			
Male	57.3	51.9	0.397
			
*Country, %*			
UK	15.6	6.2	0.103
Ireland	5.2	7.4	
Germany	13.5	12.3	
France	11.5	19.8	
Italy	34.4	30.9	
Belgium	19.8	23.5	
			
*Time since MM diagnosis, years*			
Mean (s.d.)	3.9 (3.0)	2.8 (2.5)	**0.001**
Median (Q1–Q3)	3.2 (1.9–4.9)	2.2 (1.2–3.4)	
Min–max	0.4–20.5	0.1–12.4	
			
*ECOG performance status, %*			
0	35.4	31.5	0.652
1	46.9	45.1	
2	11.5	13.6	
3	2.1	5.6	
4	1.0	0.6	
Missing	3.1	3.7	
			
*ISS, %*			
Stage I	16.7	21.0	0.831
Stage II	18.8	16.0	
Stage III	17.7	16.7	
Missing	46.9	46.3	
			
*Treatment line, %*			
Second line	84.4	93.8	**0.039**
Third line	13.5	6.2	
Missing	2.1	0.0	
			
*Additional antimyeloma drugs, %*			
Dexamethasone	92.7	95.7	0.736
Prednisone	5.2	3.1	0.505
			
*Dialysis required, %*			
Yes	0.0	5.6	**0.020**
No	97.9	94.4	
Missing	2.1	0.0	
			
*Serum creatinine, μmol/l*			
Mean (s.d.)	104.8 (62.5)	145.0 (326.8)	0.242
Median (Q1–Q3)	88.4 (71.0–106.1)	88.4 (70.7–114.0)	
Min–max	44.2-402.2	43.3-3933.8	
			
*Comorbidities, %*			
Neuropathy	26.0	26.5	0.718
Diabetes	18.8	13.0	0.267
Osteoporosis	14.6	15.4	0.330
Chronic heart failure	8.3	14.8	0.142
Urogenital disorders	10.4	13.6	0.559
Neuropathic pain	6.3	14.8	0.053
Gastrointestinal/hepatobiliary disorders	14.6	9.3	0.170
Depression	8.3	4.9	0.217
Arthritis	3.1	7.4	0.412
Chronic respiratory disorder	7.3	4.3	0.240
Visual impairment	6.3	3.7	**0.030**
Stroke	3.1	3.7	1.000
Hearing impairment	3.1	3.7	0.181

Abbreviations: ECOG, Eastern Cooperative Oncology Group; ISS, International Staging System; MM, multiple myeloma.

a Non-parametric *P*-value using *T*-test for continuous variables, Chi^2^ or Fisher's exact for categorical variables; in bold *P*-value<0.05.

**Table 2:** Description of study treatment received by patients along the study

*Variable*	*Bortezomib*		*Lenalidomide (*N=*162)*
	*Standard dose (*N=*31)*	*Low dose (*N=*57)*	
*Treatment duration, months*			
Mean (s.d.)	3.8 (1.4)	4.1 (2.1)	5.0 (3.3)
Median (Q1–Q3)	3.7 (2.4–5.1)	4.7 (2.4–5.6)	5.5 (3.0–6.3)
Min–max	1.5–6.9	0.2–10.9	0.4–29.5
			
*Cumulative dosage, mg/m*^*2*^ *or mg*			
Mean (s.d.)	25.8 (8.7)	10.8 (8.8)	2019.4 (1271.1)
Median (Q1–Q3)	24.9 (19.5–31.2)	7.8 (5.2–10.4)	1945.0 (835.0–3175.0)
Min–max	10.5–44.1	2.3–41.6	100.0–4950.0
			
*Average dosage, mg/m*^*2*^ *or mg per cycle*			
Mean (s.d.)	4.7 (0.7)	3.6 (1.7)	14.7 (6.6)
Median (Q1–Q3)	5.1 (4.2–5.2)	2.7 (2.5–4.8)	15.0 (10.0–19.1)
Min–max	2.9–5.8	1.6–7.5	2.8–25.0

**Table 3:** Baseline EORTC scores in the lenalidomide cohort and bortezomib dosage cohorts, presented as mean (s.d.)

*EORTC questionnaire*	*Domain*	*Bortezomib (*N=*96)*	*Lenalidomide (*N=*162)*
QLQ-C30	Global health status/QoL	54.6 (25.7)	54.8 (23.8)
	Physical functioning	69.1 (27.1)	63.7 (27.0)
	Role functioning	58.9 (34.5)	56.7 (36.9)
	Emotional functioning	69.6 (25.7)	70.3 (23.8)
	Cognitive functioning	79.5 (21.1)	77.1 (25.6)
	Social functioning	69.8 (31.7)	68.6 (32.6)
	Fatigue	42.6 (28.1)	43.0 (28.7)
	Nausea and vomiting	6.7 (15.0)	8.5 (18.9)
	Pain	36.1 (34.1)	39.7 (33.8)
	Dyspnoea	21.5 (26.6)	26.9 (28.6)
	Insomnia	32.6 (33.3)	27.2 (32.2)
	Appetite loss	19.9 (28.4)	21.4 (31.2)
	Constipation	19.1 (26.4)	26.5 (33.5)
	Diarrhoea	7.3 (16.3)	9.7 (21.1)
	Financial difficulties	15.6 (26.8)	13.6 (24.2)
QLQ-MY20	Body image	79.1 (30.9)	79.5 (30.1)
	Future perspective	54.3 (28.0)	52.7 (30.0)
	Disease symptoms	26.0 (22.5)	27.9 (22.8)
	Side effects of treatment	18.1 (13.5)	20.8 (15.3)
QLQ-CIPN20	Autonomic scale	11.1 (15.5)	14.0 (20.0)
	Motor scale	11.9 (13.5)	17.9 (17.6)
	Sensory scale	12.3 (15.1)	16.7 (19.2)

Abbreviations: EORTC; European Organization for Research and Treatment of Cancer; QLQ-C30, Quality-of-Life Core; QLQ-CIPN20, QLQ-Chemotherapy-Induced Peripheral Neuropathy; QLQ-MY20, QLQ-Multiple Myeloma; QoL, quality of life.
